# A quality control program for MR‐guided focused ultrasound ablation therapy

**DOI:** 10.1120/jacmp.v3i2.2584

**Published:** 2002-03-01

**Authors:** Tao Wu, Joel P. Felmlee

**Affiliations:** ^1^ Department of Radiology Mayo Clinic Rochester Minnesota 55905; ^2^ Present address: Department of Radiology Mayo Clinic Rochester Minnesota 55905

**Keywords:** quality control, thermal therapy, focused ultrasound, magnetic resonance imaging, thermal ablation

## Abstract

In this study, we propose a quality control program for MR‐guided focused ultrasound (FUS) ablation treatment to assess FUS beam positioning accuracy, FUS power delivery accuracy, MR imaging quality, and FUS ablation system safety. A total of 353 sonication points in Lucite cards were measured, the average placement errors were –0.06 mm in the SI direction and –0.04 mm in the LR direction. Temperature elevation was calculated from MR phase difference images and the measured water proton chemical shift (WPCS) temperature coefficient. WPCS temperature calibration for phantoms yielded a temperature coefficient of 0.011 ppm/°C. Sixteen experiments were conducted using six different phantoms to test the reliability of FUS power delivery. SNR and RF power calculated from phantom images were analyzed and stored at the MR console. A computer program was developed to integrate the system power delivery and the MR image quality control into one automated process. In the clinical trial at our institution, we expect this quality control program to be carried out before each patient treatment. If measured quality control values exceeds or below the preset values, a system service and retest should be conducted before the treatment.

PACS number(s): 87.61.–c, 87.54.–n

## INTRODUCTION

Focused ultrasound (FUS) ablation has been proposed as a noninvasive treatment method decades ago, and has been studied experimentally and clinically in various applications. Due in part to the lack of control and monitoring of ultrasound beam during ablation, the procedure has not been widely accepted. The recent developments in MR temperature imaging technique have made it possible to monitor the treatment process in near real time.^1^ ^–^ ^7^ Both *ex vivo* and *in vivo* animal studies have shown that it is feasible to coagulate tissue using MR‐guided FUS ablation.^3^ ^,^ ^4^ ^,^ ^8^ ^–^ ^10^ MR‐guided FUS ablation treatment is undergoing clinical trial at several sites in North America and the preliminary results are promising.^11^ ^,^ ^12^

During FUS ablation, highly focused acoustic energy causes rapid temperature elevation in target tissues, i.e., tumors, and results in tissue necrosis in seconds. The goal of the treatment is to completely coagulate target tissue volume and preserve surrounding healthy tissues. Accurate FUS beam positioning and reliable acoustic power delivery are required to achieve this goal. Currently, there is not an established quality control program for FUS ablation treatment. Considering the high‐temperature elevation involved in the procedure, and the accuracy and reliability requirement for the procedure, it is crucial to establish a quality control program to assure the safety and effectiveness of this treatment method.

In this study, we propose a quality control program for FUS ablation treatment. It includes FUS focus positioning accuracy testing, FUS power delivery testing, MR scanner imaging quality testing, and FUS system safety inspection.

## METHODS

A prototype MR‐guided FUS ablation system (Mark II, TxSonics, Inc., Dallas, TX) and a 1.5T MR‐imaging system (GE Medical Systems, Milwaukee, WI) were used in this study. The FUS system employs an eight‐element, spherical shell, air‐backed phased‐array ultrasound transducer. The eight elements were constructed in a sector‐vortex fashion;^10^ ^,^ ^13^ each of the elements has a curvature of 80 mm. The transducer has a diameter of 100 mm, a variable focal length from 60 mm to 200 mm, and was operated at a frequency of 1.5 MHz.

### A. FUS focus positioning accuracy testing

**Figure 1:**
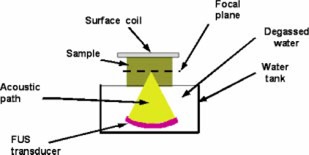
(Color) A diagram of the experiment setup. The water tank is built inside a MR table. The sample in the figure represents the Lucite card or the phantom. When the card was used, it was placed at the focal plane of the focused ultrasound transducer.

FUS focus positioning accuracy was tested in coronal plane using Lucite cards (85×53×0.4 mm).^14^ The experiment setup is shown in Fig. 1. The Lucite card was placed in a custom made holder, and water was placed in the interface between the card and the water tank to provide an acoustic path for the ultrasound beam. Multiple sets of melted spot grids were created in the cards by FUS using a treatment planning software program on the FUS workstation. The distance between neighboring spots was set to a specific length in the right‐left and in the superior‐inferior directions. On each card, the grid was created using 20‐W, 3‐sec sonications. The actual distance between two neighboring spots in each row (left‐right axis) or column (superior‐inferior axis) was measured using a digital caliper (Central Tools Inc., Cranston, Rhode Island) on the card. The difference between the prescribed distance and the actual distance was defined as placement error. When the actual distance was larger than the specific length, the error was assigned positive, if the actual distance was smaller, the error was assigned negative.

### B. FUS power delivery testing

Tissue‐simulating bovine gelatin phantoms (15% bovine powder, SIGMA Chemical Co., St. Louis, MO) were made to test the reliability of the FUS system power delivering. The phantoms were cylinders with 10 cm in diameter and 4 cm in height. Twenty‐five percent evaporated milk (in volume) was added to phantom to increase the absorption of the acoustic energy.^15^ Potassium salt (SIGMA Chemical Co., St. Louis, MO) was added to prevent bacterial invasion. The water proton chemical shift (WPCS) temperature coefficient calibration of the phantom material was conducted using previous established method.^16^ Coronal images of the phantom at the FUS focus were acquired before, during, and after a 15‐sec 80‐W FUS sonication. The images were obtained using a multiphase (14 phases) fast gradient echo sequence with 16×16cm field of view, 256×160 acquisition matrix, TR=22 ms,TE=10.7 ms, a flip angle=60°, and a 5‐in. coil. Temperature elevation at the focus was calculated from the resulting phase difference images and measured WPCS temperature coefficient.

### C. MR‐image quality control and system inspection

To assess MR‐image quality with the FUS system at treatment position (inside the MR scanner), transmitter gain, center frequency, and the SNR of the phantom images were analyzed. Both the image quality control process and FUS power delivery testing were integrated into one automated computer program. After image acquisition, the program can be executed using a single command line at the MR console.

## RESULTS

### A. FUS system positioning accuracy

**Figure 2:**
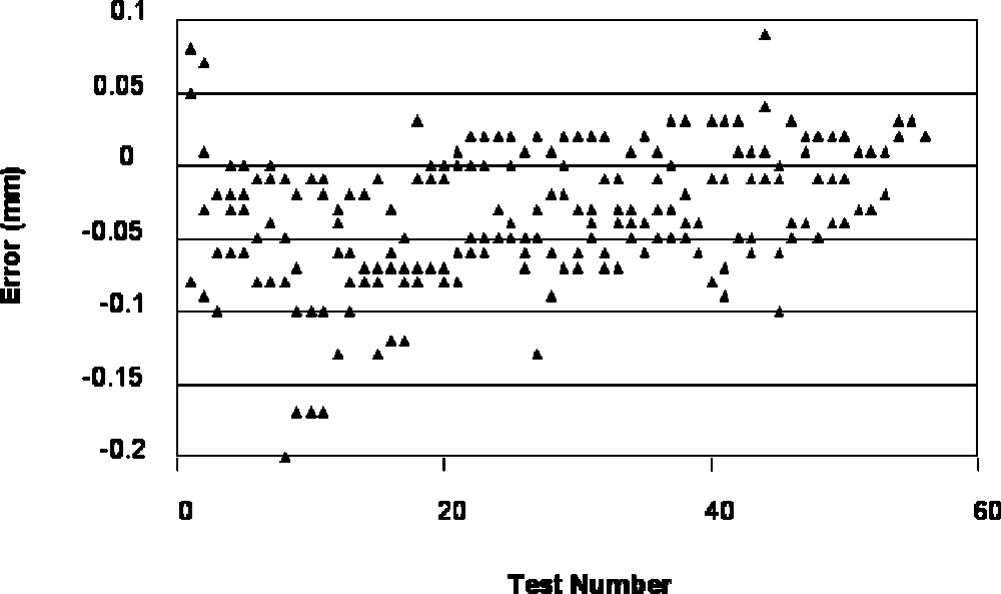
FUS system placement error measurement in the SI direction. The error value is the difference between the prescribed distance and the actual measured distance between neighboring sonication spots.

A total of 353 sonication points were measured, the overall average placement error in the superior‐inferior (SI) direction was –0.06 mm with a standard deviation of 0.076 mm. The average error in the left‐right direction was –0.04 mm with a standard deviation of 0.062 mm. Figure 2 shows the placement error measurements in SI direction.

### B. Power delivery testing

**Figure 3:**
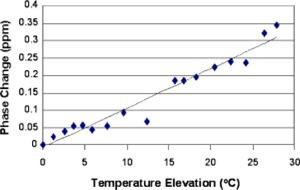
(Color) WPCS temperature calibration for the phantom.

The WPCS temperature calibration result is shown in Fig. 3. Data points shown are measured phases in a selected region of interest at different temperature. The phase values were converted into PPM in order to adjust to the magnetic field strength and imaging sequence echo time.^16^ The linear regression yielded a temperature coefficient of 0.011 ppm/°C with a standard deviation of 0.0082 ppm/°C. The correlation coefficient of the linear regression was 0.97.

**Figure 4:**
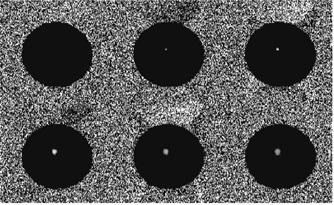
Phase difference images of a phantom during FUS sonication. The bright spot shows the phase change caused by FUS sonication.

**Figure 5:**
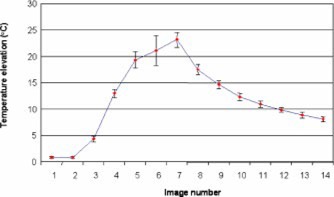
(Color) The average temperature elevation at FUS focus for 31 tests. The bar shows one standard deviation of the measurement.

Thirty‐one experiments were conducted using six different phantoms to test the reliability of FUS power delivery. Figure 4 shows phase difference images of a phantom during one FUS sonication. The bright spot in the phantom indicates the temperature elevation at the FUS focus. From top left to bottom right, these images illustrate the temperature evolution during and after FUS sonication. The average temperature changes at the focus from the 31 experiments are shown in Fig. 5. Sonication began during the acquisition of the third image and ended at seventh image.

### C. MR image quality control and system inspection

Image quality parameters, along with the temperature measurements at FUS focus, were calculated from the phantom images and stored in a text file at MR scanner. Table I shows the test results from two tests. The signal values were determined from the center of the phantom, and the noise values were determined from the four corners (air) of the images. The base temperature is the average temperature at FUS focus determined from the first two images; the peak temperature is the average temperature determined from the fifth to the eighth images; and the end temperature is the average temperature determined from the last four images. A mean value and a tolerance value for each parameter were determined based on 31 tests. Table II shows these values.

**Table I:** Quality control results generated from two tests.

Transmit gain	Signal	Noise	SNR	Center frequency	Base temperature	Peak temperature	End temperature	Study	Date
117	295	6.7	43.8	9270	0.93	20.2376	9.126	2746	9/22/01
116	286	7.1	40.2	9420	1.199	18.879	9.071	3564	10/3/01

**Table II:** Tolerance values for the quality control program. The tolerance values are set to be twice the standard deviations for the mean values.

	Transmit gain	Signal	Noise	SNR	Center frequency	Base temperature	Peak temperature	End temperature
Mean	118.00	291.24	7.77	39.47	9345.15	1.03	20.75	9.27
Tolerance	7.14	25.49	0.92	6.50	213.49	0.67	3.27	1.74

## DISCUSSION

The FUS focal spot placement accuracy is essential for a successful treatment. Previously, we have measured “absolute error” as the accuracy of sonication spot placement relative to an anatomical landmark.^14^ In this paper, we measured “relative error” as an indication of placement precision for multiple sonications. Here the placement errors of sonication spots are the differences between the prescribed and actual distances of two neighboring spots. In our current system, the absolute error is measured and corrected during patient treatment. The accuracy data acquired in this study can be used to establish safety guidelines for clinical trials. If the positioning error of the system exceeds 1 mm in any direction, system maintenance and service should be conducted before next patient treatment. A reliable power delivery of the FUS system is crucial for the effectiveness of the treatment and safety of the patient. Through numerous trials, we have constructed a phantom suitable for FUS power delivery testing. This phantom has acoustic properties close to those of human tissue,^15^ and is sensitive to heat induced by ultrasound energy. Properly stored, the phantom can be reused for months. In our experiment setup, the average base temperature is 1.03 °C, the average peak temperature is 20.75 °C, and the average end temperature is 9.27 °C. A tolerance range was assigned to each of these values (Table II). If the measured temperature value for a test falls beyond the tolerance range, a system warning will be given, and system inspection and retest should be conducted before patient treatment. We expect that the FUS system power delivery test be carried out before each patient treatment in our institution. Tissue temperature during FUS treatment is calculated from MR‐phase difference images. MR‐image quality should be maintained at high levels to ensure accurate temperature measurement during treatment. Similar to that of temperature measurements, a tolerance range was assigned to each of image quality control parameter (Table II). If any measured value of these parameters falls beyond the tolerance range, the automated program will give a system warning for inspection and retesting. We expect MR image quality control procedures be carried out before each patient treatment while the FUS system is in its treatment position. To cover other parts of the FUS ablation treatment system, we have composed a step‐by‐step treatment workbook with a system inspection list to ensure the proper implementation of system inspection, system quality control, and treatment procedure. In conclusion, we have developed a quality control program for MR‐guided FUS ablation therapy. We believe this program is important to assure safety of the patient and effectiveness of the treatment.

## ACKNOWLEDGMENT

The authors thank TxSonics Inc. (Dallas, TX) for their technical assistance.
